# Supplementary material for: Integration of over 9,000 mass spectrometry experiments builds a global map of human protein complexes
Source: Mol Syst Biol. 2017 Jun 9;13(6):932. doi: 10.15252/msb.20167490 (PMC5488662; doi:10.15252/msb.20167490)
Supplement: Supplementary file 1 — Expanded View Figures PDF [file MSB-13-932-s001.pdf]

Expanded View Figures

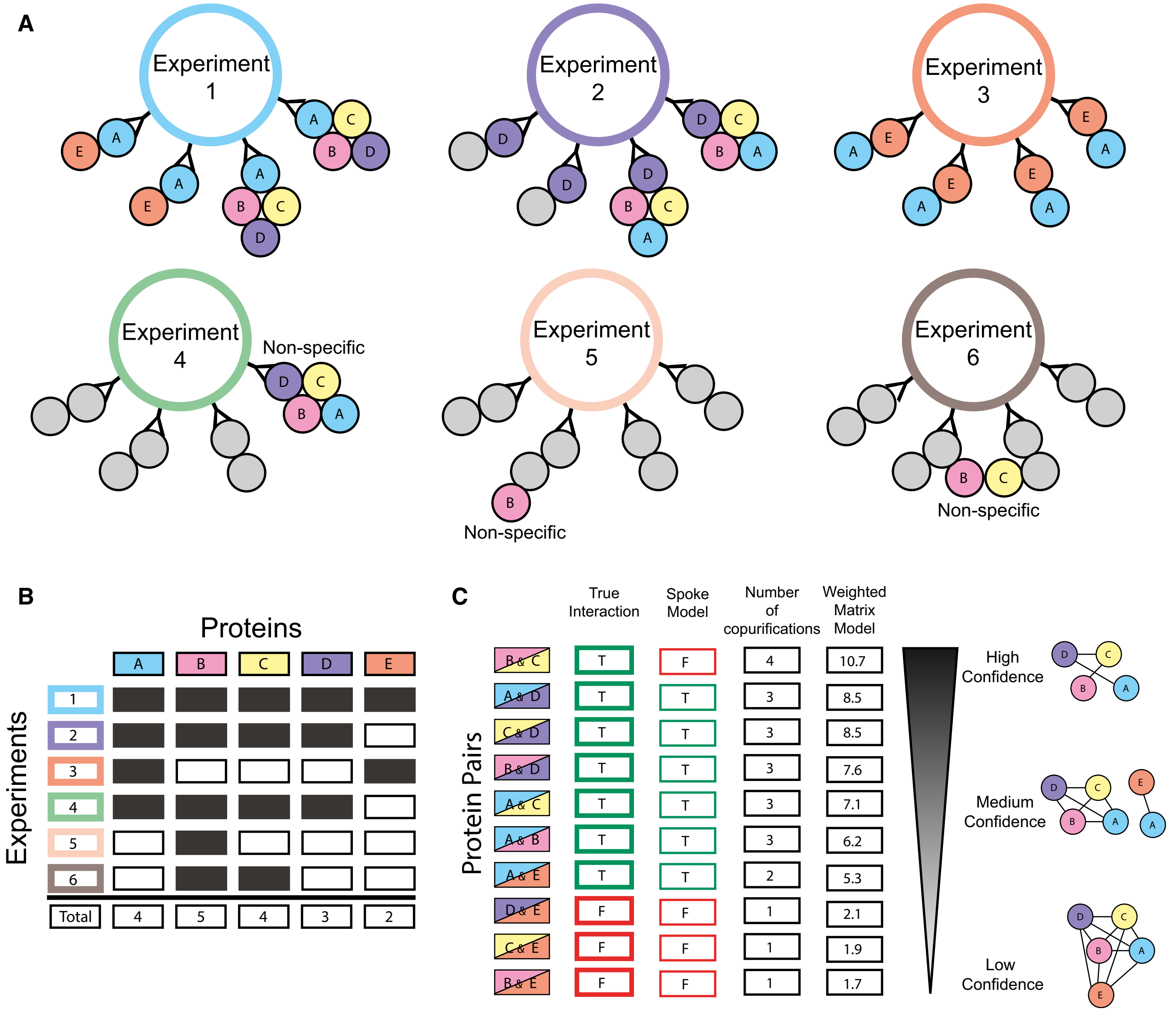

Figure EV1. Hypothetical example of co-complex interactions being scored by weighted matrix model.

A Graphical depiction of six AP-MS experiments highlighting the purification of two mutually exclusive complexes A-B-C-D and A-E. Experiments 1, 2, and 3 co-purify interactions with bait proteins A, D, and E, respectively. Experiments 4, 5, and 6 show non-specific interactions for the complexes or sub-complexes. For the purposes of this example, proteins A, B, C, D, and E are only observed in these six experiments out of a set of 50 experiments (arbitrarily defined).

B Presence–absence matrix for the six AP-MS experiments and five proteins described in (A). This matrix represents what the experimenter observes after mass spectrometry analysis.

C Calculation of weighted matrix model score for protein pairs in highlighted complexes. The *True Interaction* column represents whether the pair of proteins is co-complex or not. The *Spoke Model* column represents the predictions made by the traditional spoke model. Note the spokes model's false-negative prediction of interaction B&C. The *Number of co-purifications* column represents the number of experiments for which the pair of proteins is co-purified. The *Weighted Matrix Model* column represents the −1*log(*P*-val) of the hypergeometric test given the experimental overlap value, each protein's total number of observed experiments, and the total number of experiments (non-depicted) arbitrarily defined as 50. The panel also shows likely clusters of co-complex interactions using three levels of confidence, high, medium, and low. Note, the high- and medium-confidence networks do not show the false-positive interactions D&E, C&E, or B&E but do capture the true-positive prey–prey interaction B&C.

 © 2017 The Authors

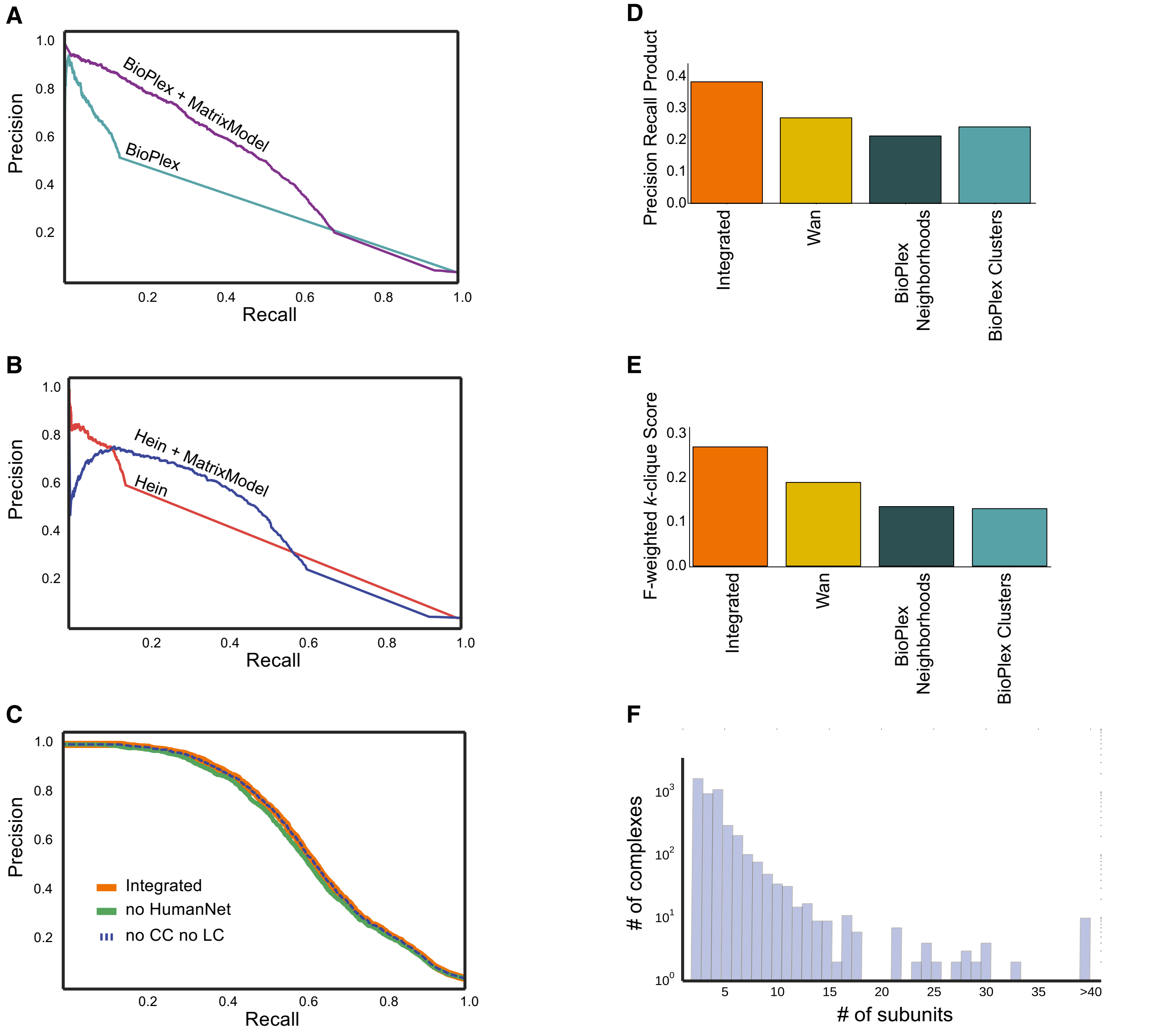

Figure EV2. Performance evaluation of networks with alternative feature sets and hu.MAP compared to leave-out set of co-complex interactions and complexes.

A Precision–recall curves calculated using leave-out set of co-complex interactions to evaluate networks trained on BioPlex only and BioPlex + weighted matrix model features. Note improvement of performance when weighted matrix model features are included.

B Precision–recall curves calculated using leave-out set of co-complex interactions to evaluate networks trained on Hein only and Hein + weighted matrix model features. Note improvement of performance when weighted matrix model features are included.

C Precision–recall curves calculated using leave-out set of co-complex interactions to evaluate networks trained on all features (integrated, orange), all features except HumanNet features SC-LC, SC-CC, CE-LC, and CE-CC (dashed blue), and all features except HumanNet (green). Note negligible performance loss when HumanNet features are excluded.

D Comparison of hu.MAP and published complex maps to leave-out set of complexes using precision–recall product measure (Song & Singh, 2009).

E Comparison of hu.MAP and published complex maps to leave-out set of complexes using *F*-weighted *k*-clique score.

F Distribution of number of subunits for complexes in hu.MAP.

© 2017 The Authors

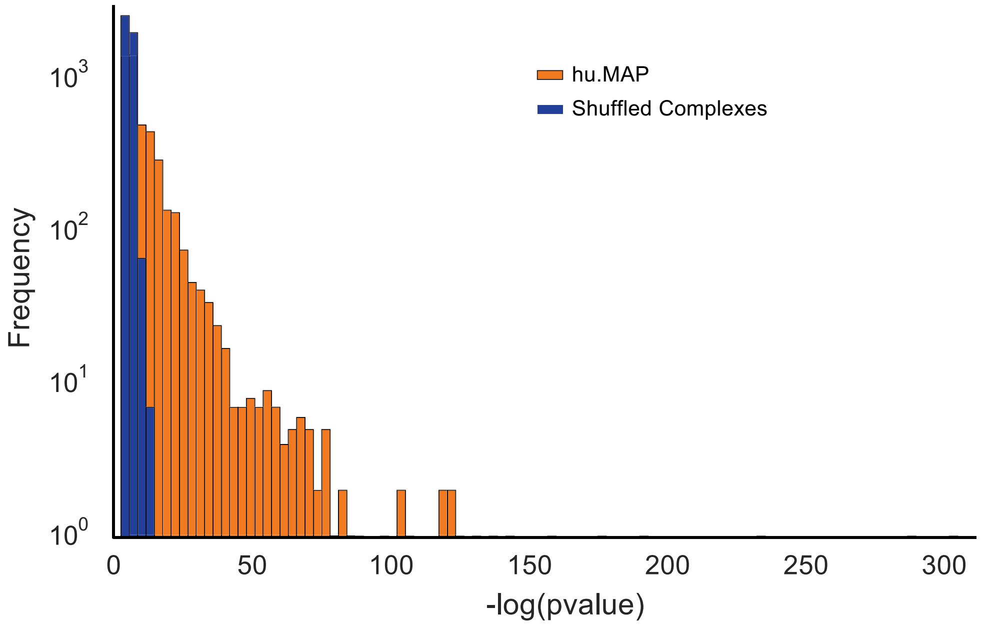

Figure EV3. Distribution of most significant annotation for each complex in hu.MAP and shuffled complexes.
We selected the largest −log(*P*-value) for each complex in hu.MAP and shuffled complexes as calculated by g:Profiler. hu.MAP has a substantially higher number of complexes with highly enriched annotations than permuted complexes. This distribution was used to calculate the false discovery rate of 5% in Fig 4B.

© 2017 The Authors

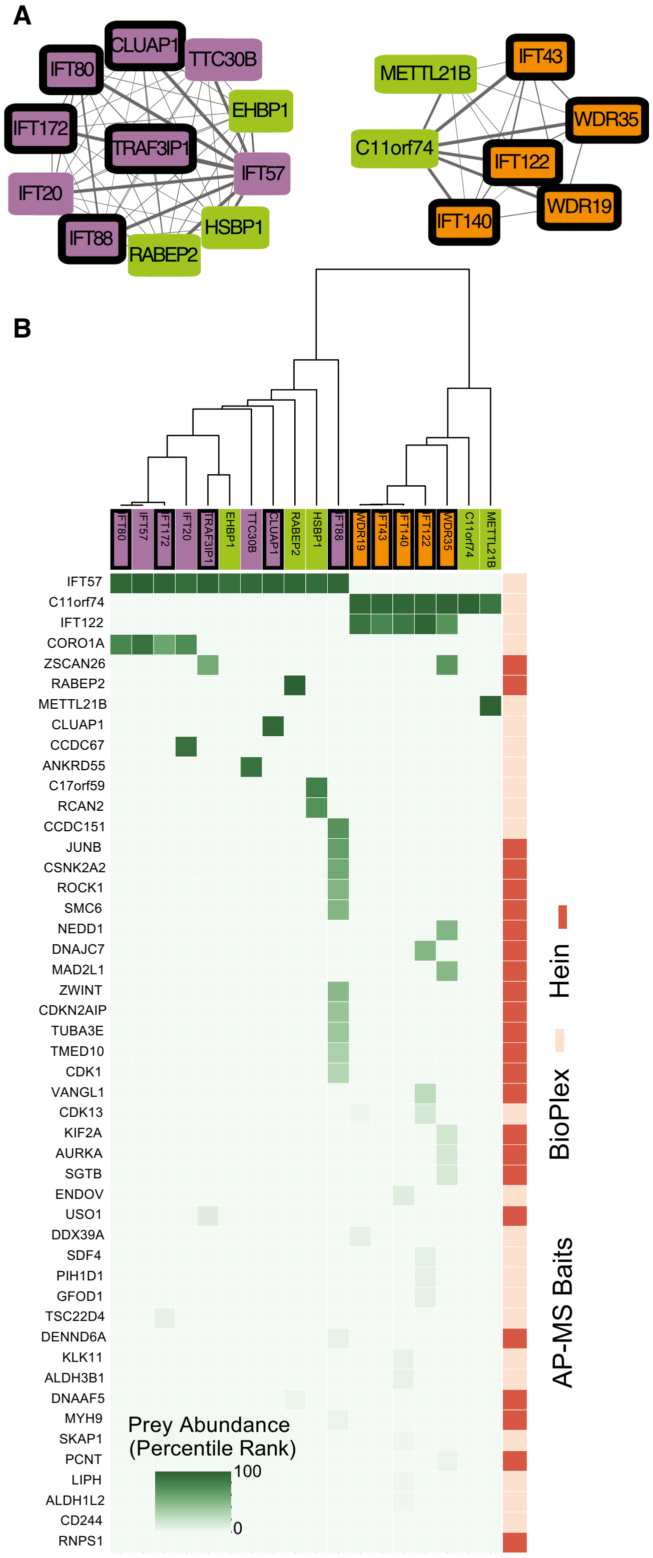

Figure EV4. hu.MAP recapitulates IFT-A and IFT-B complexes.

A Network view of IFT-A and IFT-B complexes. Node colors follow Fig 5 conventions.

B Matrix of AP-MS experiments shows IFT-A and IFT-B are well separated and supported by multiple experiments.

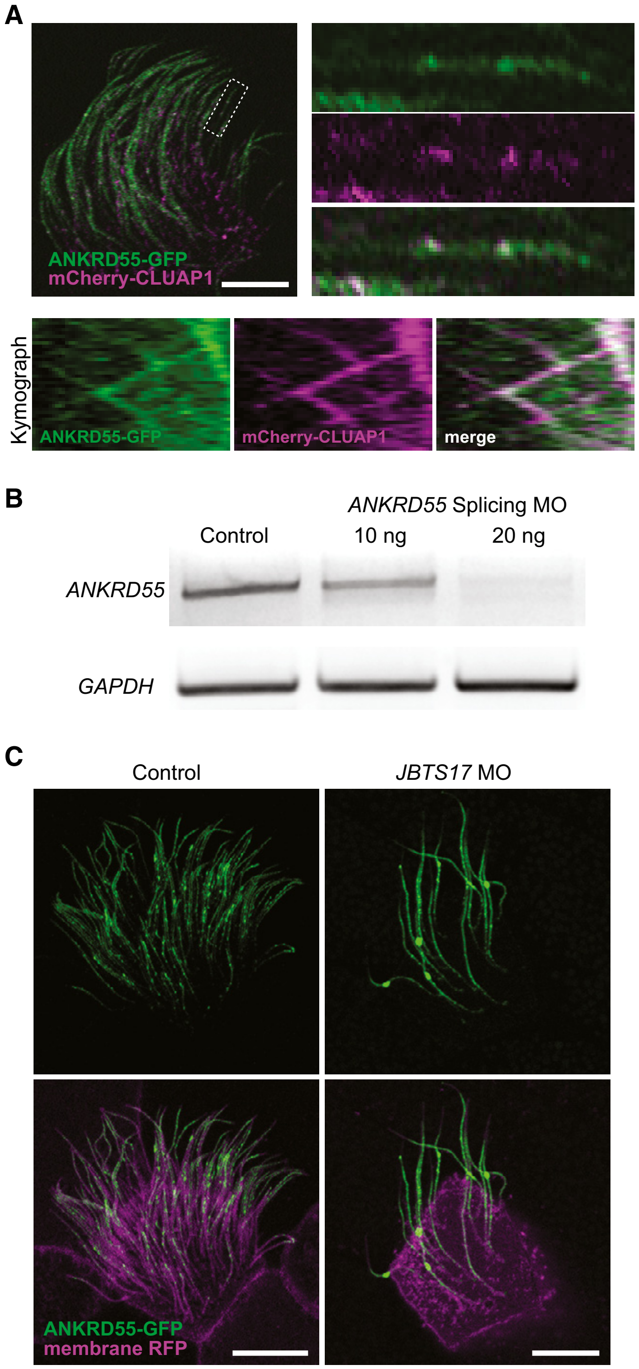

Figure EV5. ANKRD55 localization and movement along axonemes is consistent with being a component of the IFT-B particle.

A Two-color kymograph generated by co-expression of ANKRD55-GFP (green) and mCherry-CLUAP1 (magenta) reveals that ANKRD55 travels along axonemes in association with other IFT proteins. Scale bar: 10 μm. Kymograph is representative out of 22 multi-ciliated cells.

B RT–PCR demonstrates the efficiency of *ANKRD55* MO to disrupt splicing of *ANKRD55* mRNA in *Xenopus* embryos. GAPDH is used as a control.

C Morpholino knockdown of *JBTS17*, known to specifically affect IFT-B localization, results in the accumulation of ANKRD55-GFP in axonemes (green: ANKRD55-GFP, magenta: membrane RFP). Scale bar: 10 μm. Each image is representative of 18 cells from six different embryos.

 © 2017 The Authors
